# Sexual and Clinical Profile in Spanish-Speaking Individuals with Problematic Engagement in Online Sexual Activities: Comparison Between Subclinical and Clinical Groups

**DOI:** 10.1007/s10508-026-03434-0

**Published:** 2026-06-01

**Authors:** Marta García-Barba, Jesús Castro-Calvo, Cristina Giménez-García, Estefanía Ruíz-Palomino, Beatriz Gil-Juliá, Rafael Ballester-Arnal

**Affiliations:** 1 Department of Psychology and Sociology, Universidad de Zaragoza, Teruel, Spain; 2 Department of Basic, Clinical Psychology and Psychobiology, Universitat Jaume I, 12071 Castellón, Spain; 3 Department of Personality, Assessment, and Psychological Treatments, University of Valencia-Estudi General, Valencia, Spain

**Keywords:** Problematic engagement, Online sexual activities, Sexual compulsivity

## Abstract

Problematic engagement in Online Sexual Activities (OSA) has become an increasing concern, given the rapid rise of digital technologies and the consequences of this clinical picture. However, there is still no consensus regarding the nosology and diagnostic criteria of this emerging condition. Although several studies have examined the characteristics and predictors of problematic OSA, few relied on clinical samples or comprehensive diagnostic tools that encompass all existing diagnostic proposals. This exploratory study examined differences between individuals in subclinical (*n* = 51, 42.5%) and clinical (*n* = 69, 57.5%) groups of problematic OSA. We evaluated 120 individuals (95.8% men; 72.5% Spanish) aged 21 to 67 years (*M* = 35.98; *SD* = 9.96) who reported engagement in OSA and were seeking treatment for difficulties controlling sexual impulses. Participants completed an online assessment including clinical criteria drawn from several existing diagnostic frameworks, online and offline sexual behavior, and clinical variables. Individuals in the clinical group spent significantly more time and experienced more orgasms in some OSAs than those in the subclinical group; they also reported higher anxiety, depression, and sexual sensation seeking. Conversely, they spent less time in offline sexual activity. These findings suggest that individuals with clinical patterns of OSA may compensate for lower involvement in offline sexual activities by increasing their engagement in OSA. Furthermore, time spent in sexual intercourse with a steady partner, sexual sensation seeking, trait anxiety, and emotion regulation difficulties emerged as predictors of classification into the clinical group. This study clarifies differences between subclinical and clinical users and may aid in their evaluation and treatment.

**Supplementary Information:**

The online version contains supplementary material available at 10.1007/s10508-026-03434-0.

## Introduction

Problematic engagement in online sexual activities (OSA) refers to a persistent pattern of excessive and poorly controlled internet-mediated sexual behaviors, accompanied by distress and functional impairment in important areas of daily life (Chen et al., 2022; Efrati & Gola, 2018; Wéry & Billieux, 2017). Prevalence estimates of difficulties controlling OSA range from 1 to 13% (Ballester-Arnal et al., 2021; Giordano & Cashwell, 2017; Rissel et al., 2016; Wéry & Billieux, 2017). Problematic engagement in different OSAs (mainly pornography use) is a common manifestation of compulsive sexual behavior (aka, sexual addiction, hypersexuality disorder or compulsive sexual behavior disorder [CSBD]), a clinical construct that remains poorly understood and highly disputed. In this regard, it is important to note that the World Health Organization formally recognized compulsive sexual behavior disorder (CSBD) in the ICD-11 (WHO, 2019). This inclusion represents a significant step toward the clinical recognition and characterization of the condition, providing diagnostic guidelines and facilitating a common framework for its diagnosis (Stein et al., 2020). However, considerable debate persists regarding its classification, underlying mechanisms, and its boundaries with other disorders.

Regardless of the label and framework used to characterize it, there is broad consensus that compulsive sexual behavior involves (1) excessive time or effort invested on the problematic sexual behavior, (2) loss of control, (3) negative consequences in multiple areas and personal impairment, and (4) persistence in the sexual behavior despite its consequences (Carnes, 1991; Cooper et al., 1999a, 1999b; Goodman, 1992, 1998; Kafka, 2010, 2013; Rosenberg et al., 2014; World Health Organization [WHO], 2019). Additional symptoms include the use of sexual behavior as an emotional regulator and addiction-like features such as tolerance, craving, or withdrawal (Carnes, 1991; Goodman, 1992, 1998; Ince et al., 2024; Lewczuk et al., 2022; Rosenberg et al., 2014; Roza et al., 2024; Sassover & Weinstein, 2021; Wordecha et al., 2018).

### Characteristics of Individuals Exhibiting a Problematic Engagement in Online Sexual Activities

#### Sociodemographic Characteristics

The lack of consensus on diagnostic criteria makes it difficult to differentiate between individuals with CSB and those who present subclinical patterns of sexual behavior that do not yet meet clinical thresholds. To identify individuals with CSB (particularly, those exhibiting problematic engagement in OSA), experts in the field have examined sociodemographic, sexual, and clinical characteristics commonly observed in these individuals. In this sense, men are more likely to engage in OSA than women, especially pornography use (Ballester-Arnal et al., 2021; Döring et al., 2017; Weinstein et al., 2015). Sexual minority status has also been associated with increased engagement in OSA and has even been identified as a potential risk factor for problematic engagement in OSA (Træen & Daneback, 2013). Regarding age, earlier studies linked problematic OSA to middle-aged men with higher education (Daneback et al., 2006; Træen et al., 2006), but later research shows mixed results. Grubbs et al. (2019a) found an inverse relationship between age and problematic pornography use, while Ševčíková et al. (2021) observed higher scores in participants ≥ 50 compared to younger groups. Furthermore, the DSM-5 field trial found that hypersexual patients tended to be older and male compared with patients with general psychiatric conditions or substance-related disorders (Reid et al., 2012). Therefore, the role of age in problematic OSA remains unclear.

#### Characteristics of Compulsive Sexual Behavior

As for the characteristics of the OSA itself, pornography is the most common OSA among individuals with problematic engagement (Bőthe et al., 2020, 2024; Gola et al., 2020; Grubbs et al., 2017; Grubbs et al., 2024; Rissel et al., 2016; Solano et al., 2018). In addition, particular characteristics of pornography use -such as the immediate reinforcing value or the great diversity of novel content- have been shown to be potentially addictive (Flayelle et al., 2023; Ince et al., 2023, 2024; Palazzolo & Bettman, 2020; Wordecha et al., 2018). Other OSAs (e.g., webcams, online dating, sexting) also show addictive risk due to anonymity, interactivity, accessibility, and immediacy (Ballester-Arnal et al., 2021; Courtice & Shaughnessy, 2017; Shaughnessy & Byers, 2014; Van Ouytsel et al., 2020). These activities have been suggested to have addictive potential due to features such as anonymity, interactivity, easy access, the immediacy of contact with other users, and the potential of rapid escalation (Carnes et al., 2007; Griffiths, 2001; Riemersma & Sytsma, 2013). This explains why individuals with problematic engagement in OSA may exhibit problems with very different online sexual behaviors (Brand et al., 2016; Varfi et al., 2019; Wéry & Billieux, 2017), therefore, all sexual activities—not only the main ones—should be explored when assessing the clinical picture. In this line, offline sexual activities also play a very important role.

Traditionally, some researchers claimed that the use of OSA, especially in individuals with compulsive sexual behavior, served as a substitute for real-life sexual contact or to enhance unsatisfactory offline sexuality (Kuss & Griffiths, 2011), leading to the expectation of reduced offline activity. However, if problematic engagement in OSA is classified as one possible manifestation of compulsive sexual behavior (Gola et al., 2020), both online and offline sexual practices may be involved. Some studies associate OSA with more offline partners and paid sex (Studer et al., 2019; Wright, 2013), while others link it to dysfunction or dissatisfaction (Blais-Lecours et al., 2016; Landripet & Štulhofer, 2015). Therefore, it seems that compulsive sexual behavior should not be only defined on the basis of the activities that are performed, but also by how sexual activity itself is performed.

Thus, some authors have focused on studying the variables related to the performance of OSA, Several studies reported direct associations between time and frequency and problematic OSA (Ballester-Arnal et al., 2021; Bőthe et al., 2018; Chen et al., 2019), and others suggested that > 10 h per week may indicate compulsive or addictive behavior (Cooper et al., 2001; Grov et al., 2008; Voon et al., 2014; Wetterneck et al., 2012), though this varies depending on the OSA explored (Daneback et al., 2006; Grov et al., 2008). Moreover, some recreational users report equal or greater frequency and time than problematic users (Bőthe et al., 2020; Grubbs et al., 2019b; Wéry & Billieux, 2016). Thus, these measures are relevant but insufficient for diagnosis.

Other variables studied have to do with discomfort and control over online sexual activities. According to the different diagnostic proposals, experiencing discomfort with the activity and persisting in these behaviors in an uncontrolled manner seem to be key symptoms in the diagnosis of this problem (Carnes, 1991, Goodman, 1992, 1998; Kafka, 2010, 2013; Rosenberg et al., 2014; WHO, 2019). For this reason, many studies use people who experience discomfort when participating in online sexual activities as a clinical sample. Grubbs et al. (2019b), Grubbs et al. (2015) and Leonhardt et al. (2018) evidenced that there are people who feel discomfort with their consumption of OSA, mainly due to the moral incongruence between the performance of the behavior and the values or religious beliefs, which leads them to perceive their consumption as problematic and uncontrolled. Therefore, although discomfort and lack of control over the activity are important and, in many cases, essential for making a diagnosis, they should not be the only criteria to be taken into account.

In addition to this, the relationship between some clinical variables and problematic engagement in OSA has also been studied. Some psychological disorders such as depression, anxiety and even other problems such as low self-esteem or loneliness are relatively frequent among people who present a problematic engagement in OSA (Barrault et al., 2016; Borgogna et al., 2020; Levin et al., 2012; Schwartz & Southern, 2000; Wéry et al., 2020; Zlot et al., 2018;). These symptoms can appear as a consequence of consumption, as a predisposing factor, precipitant or maintainer of problematic engagement in OSA (Patterson & Price, 2012; Wéry et al., 2016; Willoughby et al., 2014). This last point would be reinforced by the use of OSA as an emotional regulator identified in people with problematic engagement in OSA (Bolshinsky & Gelkopf, 2019; Castro-Calvo et al., 2018; Wéry et al., 2016), being also one of the diagnostic criteria for hypersexuality disorder included in Kafka's (2010, 2013) proposal. However, use of cybersex as an emotional regulator also occurs in people who present subclinical consumption, even being included within a subtype called anxious-depressive, for whom cybersex functions as a strategy to regulate these symptoms (Carnes, 1991; Goodman, 1998; Kafka, 2013; Lew-Starowicz et al., 2020; Testa et al., 2024). Therefore, it is difficult to discriminate between clinical and subclinical groups on the basis of this variable.

Other lines of research have explored the relationship of some personal factors with problematic engagement in OSA. One of the most explored variables is sensation seeking and, specifically, sexual sensation seeking, which has also been related to hypersexuality and particularly to problematic use of the Internet for sexual purposes (Chen et al., 2018, 2019; Walton et al., 2017). This variable, together with impulsivity, has been related to sexual risk behaviors (Gil-Llario et al., 2016) and even compulsive sexual behavior (Antons & Brand, 2018; Charnigo et al., 2013; Chen et al., 2018; Levi et al., 2020).

#### Different Online Sexual Activities Consumption Profiles

Taking into account the characteristics that may be associated with problematic engagement in OSA, some authors have sought to identify different profiles of OSA use. One of the first studies in this field was conducted by Cooper et al. (1999a, 1999b). Cooper et al. identified three profiles (recreational, at-risk, and addiction) based on symptoms such as frequency of use, time spent, and experienced consequences. This classification has been used in numerous studies (Rosser et al., 2014; Wetterneck et al., 2012), facilitating the identification of risk factors and/or clinical characteristics of individuals with problematic engagement in OSA. Nevertheless, a major methodological limitation of this initial classification was that it was derived from the authors’ clinical experience and observations, without the support of any analytical approach for group identification (e.g., cluster or latent class analysis). This limitation has prompted subsequent researchers to employ more rigorous methodologies to identify profiles that better differentiate between clinical, subclinical, and non-clinical populations. In this regard, Vaillancourt-Morel et al. (2017) identified three profiles of pornography consumers, very similar to those proposed by Cooper et al., (1999a, 1999b). However, these authors distinguished between recreational, compulsive, and highly distressed non-compulsive groups. This last group comprised individuals who, although they did not exhibit a compulsive sexual profile, experienced disruptions in their sexuality (e.g., lower sexual satisfaction). Hernández-Mora et al. (2023) also identified three profiles of pornography users from an addictive perspective (non-problematic, at-risk, and problematic). These authors found a higher prevalence of symptoms such as tolerance, withdrawal, loss of control, and time spent among problematic users; however, they did not find differences in symptoms such as anxiety. Bőthe et al. (2020) also identified three profiles of pornography users: nonproblematic low-frequency pornography use, nonproblematic high-frequency pornography use (NPHFU), and problematic high-frequency use (PHFU), highlighting that factors such as hypersexuality, depression, susceptibility to boredom, and lower self-esteem, among others, were specifically associated with PHFU and not with NPHFU. Subsequent studies have identified a larger number of profiles, including several risk profiles, based on factors such as moral incongruence or the presence of certain diagnostic criteria (Bőthe et al., 2025; Zarate et al., 2025).

### The Present Study

These studies provide valuable insights into problematic OSA, but most of them only include people with problematic use without differentiating clinical and subclinical profile. This distinction is crucial for identifying risk factors, treatment needs, and prevention strategies, as well as for refining theoretical models and diagnostic frameworks for OSA. In addition, many of those that include a clinical sample do not carry out an exhaustive assessment, which could lead to false diagnoses. Moreover, most studies primarily use samples of pornography consumers, without considering other OSA. Although research on problematic pornography use is very valuable and has greatly advanced our understanding of sexual compulsivity, other sexual behaviors remain underexplored, contributing to inconsistent findings and complicating differentiation between profiles. Taking into account the methodological limitations found in previous studies, as well as discrepancies in some aspects of this problem, the present study aims to explore the differences between individuals with subclinical and clinical profiles of problematic cybersex use who engage in OSA. Specifically, we employed the SSCI-CSB interview (Castro-Calvo et al., 2026) to identify the presence/fulfilment of different existing diagnostic proposals for compulsive sexual behaviour disorder, sexual addiction, and hypersexual disorder. This approach facilitates the differentiation of subjects with a clinical profile from those with a subclinical profile. In addition, different aspects of their sexuality will be explored in depth, as well as the presence of other clinical symptoms.

## Method

### Participants

For this study we recruited a sample of 120 participants ranging from 21 to 67 years old (*M* = 35.98; *SD* = 9.96) who were seeking treatment for problems controlling their sexual impulses. By gender, 115 were male (95.8%) and the rest (4.2%) were female. There were no significant differences by age between the groups (*t* = .34; *p* = .731). Regarding their relationship status, 70.8% had a steady partner at the time of the evaluation, 25.8% were single and 3.3% had casual relationships. In addition, with regard to sexual orientation, 75.8% identified as heterosexual, 13.3% as bisexual and 10.8% as homosexual. The greater part of participants (72.5%) had Spanish nationality and lived in Spain at the time of the evaluation, while 2.4% had Spanish nationality but lived in another European country. The remaining participants (25.1%) were born in a Spanish-speaking country, including Colombia, Mexico, Argentina, among others. All of them were residing in their country of origin at that time.

In terms of educational level, 64.9% had university studies (41.6% undergraduate/graduate degree; 23.3% master's or doctorate; 23.3% had completed professional training; 10% had secondary studies, while 1.7% had only primary studies). Among them, 74.2% were in active employment at the time of the evaluation, 14.2% were students and 11.6% were unemployed or retirees. Finally, regarding religious beliefs, 55.8% identified as atheist/agnostic, 28.3% as non-practicing believers and 15.8% as practicing believers. No statistically significant differences were found between the groups in the distribution of these variables (*p* between .133 and .908).

### Procedure

Data acquisition was carried out between 2019 and 2024. The initial 150 study participants requested free treatment for problematic engagement in OSA through our website created for this project (https://adiccionalsexo.uji.es). To ensure the clinical relevance of self-reported symptoms, all participants were thoroughly evaluated. This assessment consisted of two batteries of questionnaires that participants completed online, in addition to a comprehensive evaluation of 3 sessions with a therapist (via video call). These batteries were used to analyze problematic and non-problematic online and offline sexual behavior, the presence of comorbidities or excluding diagnoses, as well as other clinical variables. The first online battery was completed before the first contact between the evaluator and the participant, while the second was completed online between the first and second video call. Six clinicians (two men and four women) participated in patients’ assessment and treatment. All the therapists were doctoral-level psychologists with a strong background on sex therapy and working at a university-based sexual disorder specialty clinic [masked for review]. Each session lasted approximately an hour and a half, during which the Semi-Structured Clinical Interview for the Assessment of Compulsive Sexual Behavior (SSCI-CSB) was completed (Castro-Calvo et al., 2026). Participants were randomly assigned to one of these therapists. The inclusion criteria focused primarily on the presentation of a clinical or subclinical [addiction or risk in the original scale] profile on the Internet Sex Screening Test (Ballester-Arnal et al., 2010; Delmonico, 1997). In total, 24 people were eliminated for this reason. Furthermore, we also ruled out people who had psychiatric diagnoses and/or medical conditions that could explain this problematic pattern such as the presence of Tourette's syndrome (*n* = 1), bipolar disorder or schizophrenia (*n* = 4), and Parkinson's disease (*n* = 1). In order to complete both the initial assessment battery and the rest of the evaluations also the inclusion criteria included being over 18 years of age and speaking Spanish.

The 120 participants were divided into two groups: subclinical and clinical. People in the clinical group scored greater than or equal to 9 in the Internet Sex Screening Test (Ballester-Arnal et al., 2010; Delmonico, 1997). They also complied with at least one of the existing diagnostic proposals for sex or cybersex addiction/compulsivity/hypersexuality. These proposals are those of Carnes (1991), Goodman (1992, 1998), Kafka's (2010), and ICD-11 mentioned in the introduction section. All the criteria of these proposals are included in the Semi-Structured Clinical Interview for the assessment of Compulsive Sexual Behavior (SSCI-CSB) (Castro-Calvo et al., 2026) or in the ICD-11. In this study, 14.5% of the participants met all four diagnostic proposals, 31.9% met three of the diagnostic proposals, 24.6% met two of the diagnostic proposals and 29% met one of the diagnostic proposals. Of these, the diagnosis of addiction according to Carnes (1991) is the most prevalent (76.8%), followed by Kafka's hypersexuality (Kafka, 2010) (66.7%), compulsive sexual behavior disorder in ICD-11 (WHO, 2019) (60.9%) and compulsive Goodman's criteria (1992; 1998) (27.5%). Participants in the subclinical group had at least a risky profile according to the Internet Sex Screening Test (Ballester-Arnal et al., 2010; Delmonico, 1997) (scores between 9 and 18) but did not meet any of the addiction-compulsivity-hypersexuality diagnosis (clinical criteria).

### Measures

*Sociodemographic information*: Different sociodemographic characteristics were explored through a questionnaire developed ad hoc. These include perceived sexual orientation (heterosexual, homosexual, bisexual, pansexual, asexual or others); gender (I identify as: male, female or non-binary); age; country of residence and nationality; and relationship status (single, in a stable relationship or with sporadic partners). We also included items about the educational level (degree or bachelor's degree, master's or doctorate, professional formation, secondary education, elementary education), current employment status (active, studying, unemployed or pensioner/retired) and religious beliefs (atheist/agnostic, non-practicing believer, practicing believer).

*Internet Sex Screening Test* (Delmonico, 1997; Spanish adaptation by Ballester-Arnal et al., 2010*)*: This 25-item dichotomous (true = 1/false = 0) instrument is used to explore online sexual behavior. It also allows discriminating among three cybersex consumption profiles: recreational profile (scores between 0 and 8); risk profile (scores between 9 and 18) and addiction profile (scores between 19 and 25). Its Spanish adaptation (Ballester-Arnal et al., 2010) was validated in a sample of 1239 young people. This validation presented good internal consistency (*α* = 0.88), and good test–retest stability (*r* = 0.82). Like its original version, this translation also revealed the existence of five factors: online sexual compulsivity, online sexual behavior-isolated, online sexual behavior-social, online sexual spending, and interest in online sexual behavior. Internal consistency obtained from our data is *α* = .69..

*The Semi-Structured Clinical Interview for the assessment of Compulsive Sexual Behavior (SSCI-CSB)* (Castro-Calvo et al., 2026): Semi-structured interview designed to comprehensively explore online and offline sexual behavior and the symptoms and clinical criteria of cybersex addiction-sexual compulsivity-hypersexuality agreed upon by various authors (Carnes, 1991; Goodman, 1992, 1998; Kafka, 2010, 2013; Rosenberg et al., 2014; WHO, 2019). This interview consists of three parts, but for the purposes of this study only the first two were employed.

The first part explores online sexual activities (viewing pornographic images or movies, flirting and sexual advances towards other users, chatting for sexual purposes with other users via text, webcam sexual contact with another user, finding a sexual partner, finding a romantic partner, purchasing sexual material, contacting sex workers, and other activities) and offline sexual activities (masturbation without using online sexual materials, sexual intercourse with a stable partner, sexual intercourse with sporadic partners–in the case of not having a stable partner-, sexual intercourse with persons other than a stable partner–infidelity–and sexual intercourse with sex workers) according to the performance of the behavior during the last 6 months. All these activities are assessed in terms of weekly time spent on each sexual activity, weekly orgasms experienced with each of the sexual behaviors, the degree of discomfort produced by the behavior itself (0 = not at all, 10 = a lot) and the degree of perceived control over the sexual behavior (0 = no control, 10 = absolute control).

The second part of the interview includes the criteria for the different diagnostic proposals. These are: Excessive investment of time and effort in sexual activities; persistent inability to resist the temptation to engage in excessive sexual behavior; Persistent desire or unsuccessful efforts to control or interrupt sexual behavior; Performance of the sexual behavior with greater frequency or duration than planned; Occurrence of behavioral consequences of the excessive sexual behavior; Persistent performance of the behavior despite the consequences; Significant reduction of social, academic and work activities; Omission of responsibilities (social, occupational, family, academic…); Use of sex to regulate dysphoric moods; Occurrence or aggravation of sexual behavior in the face of stressful life events; Tolerance, Craving and Withdrawal symptoms, Increasing sense of tension before initiating sexual behavior; Relief and release once the sexual behavior has been performed and negative feelings (guilt, shame, etc.) soon after performing the sexual behavior. In addition, the differential diagnosis and being over 18 years of age are also included. All of them are evaluated according to two time points: during the last 6 months and prior to these last 6 months through open-ended control questions. For the assessment of each diagnostic symptom, the interview includes specific control questions and operational definitions that guide the clinician in coding: 0 (absence of the symptom), 1 (subthreshold symptom) or 2 (clinical symptom). Patients showing features of a particular symptom but to a lesser extent or lower severity to consider that they totally met the criterion were scored as subthreshold. In addition, the patient assesses the degree of discomfort (0 = not at all, 10 = very much) that each of the symptoms causes.

In the third part of the interview, information is collected on the onset and clinical course of the problem: age of onset of consumption and problematic use; control and discomfort at different relevant moments in the development of the addiction; perception of the severity of the problem; search for information and/or consultation with a professional, family member, friend, etc.; diagnosis if a professional has been contacted; and treatments received. Most of the answers to these questions are dichotomous (e.g., Yes/No, Episodic/continuous course, Gradual/acute onset, etc.) while others are open-ended (for example, diagnosis received, type of treatment, etc.).

*The Beck Depression Inventory (BDI-II)* (Beck et al., 1996; Spanish adaptation by Sanz et al., 2003): Self-report consists of 21 items with a 4-point Likert response (from 0 [absence of the symptom] to 3 [maximum severity] in the direct items) designed to detect and evaluate the severity of depression. Different symptoms characteristic of depression (e.g., sadness, loss of interest, etc.) are collected in this scale. The total score on the scale ranges from 0 to 63 points. A score between 0 and 13 would indicate the absence of depressive symptoms while the remaining scores would indicate different degrees of depressive symptomatology: mild depression (between 14 and 19 points), moderate depression (between 20 and 28 points) and severe depression (between 29 and 63 points). Its Spanish adaptation shows an internal consistency of .87, similar to that obtained in the present study (*α* = .89)..

*The Barratt Impulsivity Scale (BIS-11)* (Barratt & Patton, 1983; Patton et al., 1995; Spanish adaptation by Oquendo et al., 2001): This scale consists of 30 items that explore different dimensions of impulsivity: Cognitive Impulsivity (Attention), Motor Impulsivity and Unplanned Impulsivity. Each item consists of four response options (1 = rarely/never, 2 = occasionally, 3 = often; 4 = always or almost always). Scores range from 30 to 120. A score higher than 72 indicates high impulsivity, between 52 and 71 indicates normal impulsivity and scores below 51 indicate low impulsivity. Its Spanish adaptation presents a high internal consistency (α = .80) similar to that obtained in the present study (α = .82)..

*Difficulties Emotion Regulation Scale (DERS)* (Gratz & Roemer, 2004; Spanish adaptation by Hervás & Jódar, 2008): This scale evaluates different aspects of emotional dysregulation through 28 items with a 5-point Likert scale (1 = Almost never [0–10%], 2 = Sometimes [11–35%], 3 = Half the time [36–65%], 4 = Most of the time [66–90%], 5 = Almost always [91–100%]). It is composed of 5 factors: nonacceptance of negative emotional responses, difficulties engaging in goal-directed behaviors, difficulties controlling impulsive behaviors, lack of emotional awareness, and lack of emotional clarity. The original scale showed high internal consistency (α = .93) identical to that obtained in this study (α = .93). A high score on this scale indicates possible difficulties in emotional regulation.

*State-Trait Anxiety Inventory (STAI)* (Spielberger et al., 1970; Spanish adaptation by Seisdedos, 1982): Scale consists of 40 items divided into two subscales of 20 items each: the anxiety-state scale (transitory emotional condition), and the anxiety-trait scale (tendency to anxiety relatively stable). Responses were on a four-point scale ranging from not at all (0) to very much (3). The score ranges for each subscale oscillate between 0 and 60, and has a differential rating for men and women. Scores equal to 19 in both subscales in men and equal to 21 in the anxiety-state scale and 24 in the anxiety-trait scale in women, place the person in the 50th percentile. Its psychometric properties in the Spanish population are good (between 0.90 and 0.94) (Fonseca-Pedrero et al., 2012; Guillén-Riquelmeé & Buela-Casal, 2011). The internal consistency in the present study is .94 for the anxiety-state subscale and .91 for the anxiety-trait subscale.

*Rosenberg Self-Esteem Inventory (RSEI)* (Rosenberg, 1979; Spanish adaptation by Martín-Albo, et al., 2007): This scale, designed to explore self-esteem, is made up of 10 items with a 4-point Likert-type response format (0 = strongly agree, 3 = strongly disagree). It has a good validity and reliability (α = .87 and test–retest reliability of .85). Although there is no strict rating, Rosenberg (1979) indicates that scores below 25 would indicate low self-esteem. The internal consistency with our data is .91.

*Sexual Sensation Seeking Scale (SSSS)* (Kalichman & Rompa, 1995; Spanish adaptation by Ballester-Arnal et al., 2018): This scale is composed of 11 items with 4-point Likert-type response options (1 = not at all characteristic of me; 4 = very characteristic of me), its internal consistency being .87. The minimum score is 11 points and the maximum is 44, this measurement being positive: the higher the score, the greater the search for sexual sensations. In the present study the internal consistency is .83.

### Data Analysis

Statistical analyses were performed with the SPSS statistical package (version 27.0) and the G*Power 3.1 program (used to calculate the effect size). For the analysis of the differences between the groups in the time spent in online and offline sexual activities, a sum of the time spent in each OSA and each offline sexual activity, respectively, was performed. Subsequently, Student's t statistic was used to analyze the differences in the means between the two groups.

Moreover, the most frequent online and offline sexual activities were comprehensively analyzed. For this purpose, the activities carried out by more than 10% of the participants were selected (see Supplementary Table 1 and Supplementary Table 2 for additional details on the variables not included in the main analysis). These activities were: viewing pornographic images or movies, flirting and sexual advances towards other users, chatting for sexual purposes with other users via text, webcam sexual contact with another user, finding a sexual partner, masturbation without using online sexual materials, sexual intercourse with a stable partner, sexual intercourse with sporadic partners. For these activities, the differences between the groups were analyzed according to the percentage of people who carried out this activity using the chi-square statistic. The differences in time spent, orgasms experienced, degree of control and degree of discomfort were measured using the nonparametric Mann–Whitney U statistic (due to the small sample size; in all variables the subclinical group was equal to or less than 50). Next, differences in clinical variables related to problematic engagement in OSA (Internet Sex Screening Test and its subscales) and other clinical variables (sexual sensation seeking, self-esteem, trait-state anxiety, depression, impulsivity and difficulties in emotional regulation) were analyzed. Student's* t* statistic was used for these differences, since the sample size for these variables was adequate and the criteria of normality and homogeneity of variances were met. Magnitude of the differences for tests performed with the chi-square statistic was calculated using Cramer's *V*, while Cohen's *d* was used to calculate the effect size for continuous variables (analyses performed with the Mann–Whitney *U* test and Student's *t* test). For Cramer's *V*, effect sizes around .10 are considered small, around .30 moderate and close to .50 large, while for Cohen's *d* effect sizes above .20 are small, around .50 medium and 0.80 or higher are considered large (Ellis, 2010).

Finally, seven binary logistic regressions were performed since our dependent variable is categorical (belonging or not to the clinical group). Moreover, due to the n of our study the number of independent variables (IV) introduced in each analysis is not recommended to be higher than 6–7 variables. Considering this limitation, the models were specified as follows: Model 1: time spent on OSAs in general and time spent in different OSA: viewing pornographic images or films, flirting and sexual advances towards other users, chatting for sexual purposes with other users via text, sexual contact via webcam with another user, search for a sexual partner; Model 2: time spent on offline sexual activities in general and time spent in different offline activities: masturbation without online sexual material, sexual intercourse with a steady partner, sexual intercourse with sporadic partners; Model 3: orgasms experienced in the different OSAs mentioned in Model 1; Model 4: orgasms experienced in the different offline sexual activities mentioned in Model 2; Model 5: discomfort and control over the different OSAs mentioned in Model 1; Model 6: discomfort and control over the different offline sexual activities mentioned in Model 2 and Model 7: psychological traits (sexual sensation seeking, anxiety-trait and state; self-esteem; depressive symptoms; impulsivity and difficulties in emotional regulation).

The method used was stepwise backward because, in these cases where the subject matter is very new and the researchers do not know which variables might have more weight in the model, running a stepwise analysis and fitting the selected model provides a better prediction than no model at all (Olusegun et al., 2015). Although we acknowledge the limitations of stepwise procedures (e.g., risk of capitalizing on chance and unstable parameter estimates) (Babyak, 2004; Steyerberg et al., 1999; Whittingham et al., 2006), they were used here for exploratory purposes. To address these limitations and assess the robustness of our findings, we also examined zero-order correlations among all independent variables (see Supplementary Table 3) and conducted a hierarchical logistic regression including the most relevant predictors, selected based on their theoretical relevance, following the recommendations of the events by variable (Moons et al., 2014; Pavlou et al., 2015) (see Supplementary Table 4).

## Results

### Online Sexual Activities

When analyzing the overall time spent by participants in these activities, we found statistically significant differences between the two groups (*t* = − 2.57; *p* = .011; *d* = .560). Individuals in the clinical group spend an average of 21.97 h/week on these activities (*M* = 1318.30 min; *SD* = 1520.10 min) while the subclinical group spends an average of 12.43 h/week (*M* = 745.98 min; *SD* = 523.07 min). When exploring OSAs comprehensively, we found that some activities such as viewing pornographic images or films or flirting and searching for a sexual partner were quite frequent (more than 20% of participants in each group performed these activities). In contrast, behaviors such as Search for a romantic partner, purchase of sexual material and contact with sex workers were performed by less than 10% of the participants in each group. For this reason, only those that were carried out by a considerable percentage of participants were analyzed exhaustively (more than 10%).

As shown in Table 1, when comprehensively analyzing online sexual activities, we found some differences between the two groups. In general, people in the clinical group perform more OSA, spend more time on these activities, experience more orgasms, and perceive greater discomfort and less control; however, only some of these differences were statistically significant. Individuals in the clinical group spend significantly more time per week than the at-risk or subclinical group in viewing pornographic images or films (*z* = − 2.09; *p* = .036) and in flirting and sexual advances towards other users (*z* = − 1.99; *p* = .047). In addition, individuals in the clinical group had more weekly orgasms in activities such as chatting for sexual purposes with other users via text (*z* = − 2.03 *p* = .042) and sexual contact via webcam with another user *(z* = -2.24; *p* = .025) compared to people in the subclinical group. All these differences have a moderate effect size (*d* between .527 and .767) except for differences in time spent viewing pornographic images or films that are small in size (*d* = .369).

**Table 1:** Differences between subclinical and clinical (problematic) groups in online sexual activities

	Subclinical group (n = 51)	Clinical group (n = 69)	Student *t,* U of Mann–Whitney or *χ*^2^	Effect size
Minutes online/week [M(SD)]	745.98 (523.07)	1318.30 (1520.10)	*t* = -2.576 (*p* = .011)	*d* = .560
Online Sexual Activities				
Viewing pornographic images or films	98% (n = 50)	100% (n = 69)	*χ*^2^ = 1.36 (*p* = .243)	*V* = .107
Time (minutes/week) [*M*(SD)]	323.04 (292.14)	486.30 (591.46)	*z* = -2.09 (*p* = .036)	*d* = .369
Orgasms (orgasms/week) [*M*(SD)]	5.43 (3.48)	6.15 (4.43)	*z* = -.75 (*p* = .451)	*d* = .182
Degree of discomfort (0–10) [*M*(SD)]	6.17 (2.93)	6.50 (2.68)	*z* = -.46 (*p* = .647)	*d* = .117
Degree of control (0–10) [*M*(SD)]	3.80 (2.35)	3.74 (2.30)	*z* = -.12 (*p* = .903)	*d* = .025
Flirting and sexual advances towards other users	60.8% (n = 31)	71% (n = 49)	*χ*^2^ = 1.38 (*p* = .240)	*V* = .107
Time (minutes/week) [*M*(SD)]	225.65 (179.56)	351.00 (297.21)	*z* = -1.99 (*p* = .047)	*d* = .527
Orgasms (orgasms/week) [*M*(SD)]	2.17 (2.07)	3.09 (2.74)	*z* = -1.35 (*p* = .178)	*d* = .378
Degree of discomfort (0–10) [*M*(SD)]	6.87 (2.83)	7.42 (2.69)	*z* = -1.12 (*p* = .265)	*d* = .199
Degree of control (0–10) [*M*(SD)]	4.39 (2.63)	3.42 (2.81)	*z* = -1.71 (*p* = .088)	*d* = .356
Chatting for sexual purposes with other users via text	62.7% (n = 32)	72.5% (n = 50)	*χ*^2^ = 1.28 (*p* = .258)	*V* = .103
Time (minutes/week) [*M*(SD)]	249.35 (200.23)	344.17 (293.03)	*z* = -1.30 (*p* = .193)	*d* = .377
Orgasms (orgasms/week) [*M*(SD)]	1.97 (1.62)	3.51 (3.16)	*z* = -2.03 (*p* = .042)	*d* = .644
Degree of discomfort (0–10) [*M*(SD)]	7.61 (1.73)	7.97 (2.15)	*z* = -1.48 (*p* = .138)	*d* = .179
Degree of control (0–10) [*M*(SD)]	4.14 (2.15)	3.35 (2.83)	*z* = -1.39 (*p* = .166)	*d* = .314
Sexual contact via webcam with another user	45.1% (n = 23)	58% (n = 40)	*χ*^2^ = 1.95 (*p* = .163)	*V* = .127
Time (minutes/week) [*M*(SD)]	183.04 (215.46)	346.05 (412.54)	*z* = -1.38 (*p* = .168)	*d* = .495
Orgasms (orgasms/week) [*M*(SD)]	2.04 (1.67)	4.11 (3.73)	*z* = -2.24 (*p* = .025)	*d* = .767
Degree of discomfort (0–10) [*M*(SD)]	8.02 (2.09)	8.14 (2.34)	*z* = -.46 (*p* = .646)	*d* = .054
Degree of control (0–10) [*M*(SD)]	3.18 (1.77)	3.00 (2.59)	*z* = -1.48 (*p* = .138)	*d* = .081
Search for a sexual partner	23.5% (n = 12)	39.1% (n = 27)	*χ*^2^ = 3.25 (*p* = .071)	*V* = .165
Time (minutes/week) [*M*(SD)]	212.50 (185.57)	482.67 (952.52)	*z* = -.13 (*p* = .895)	*d* = .393
Orgasms (orgasms/week) [*M*(SD)]	1.75 (1.71)	2.44 (2.87)	*z* = -.35 (*p* = .729)	*d* = .292
Degree of discomfort (0–10) [*M*(SD)]	8.00 (2.55)	6.89 (2.64)	*z* = -1.35 (*p* = .177)	*d* = .427
Degree of control (0–10) [*M*(SD)]	5.08 (2.50)	3.83 (3.15)	*z* = -1.29 (*p* = .209)	*d* = .439

### Offline Sexual Activities

As with OSA, offline sexual activities performed by less than 10% of the participants were also not extensively analyzed (Sexual infidelity and Sexual intercourse with sex workers). When evaluating the rest of the activities exhaustively (Table 2), statistically significant differences were only found in the time spent in two of the sexual activities. People in the subclinical group spend significantly more time engaging in masturbation without online sexual material *(z* = -3.97; *p* < .001; *d* = .426) and in sexual intercourse with a steady partner *(z* = -2.49; *p* = .012; *d* = .409) than those in the clinical group. However, no statistically significant differences were found in the total amount of time spent on offline sexual activities.

**Table 2:** Differences between subclinical and clinical (problematic) groups in offline sexual activities

	Subclinical group (n = 51)	Clinical group (n = 69)	Student *t,* U of Mann–Whitney or *χ*^2^	Effect size
Minutes offline/week [M(SD)]	129.49 (158.25)	113.14 (161.52)	-.555 (*p* = .580)	*d* = -102
Offline Sexual Activities				
Masturbation without online sexual material	49% (n = 25)	58% (n = 40)	*χ*^2^ = .95 (*p* = .331)	*V* = .089
Time (minutes/week) [*M*(SD)]	74.13 (57.10)	43.84 (85.09)	*z* = -3.97 (*p* < .001)	*d* = .426
Orgasms (orgasms/week) [*M*(SD)]	2.45 (1.68)	4.26 (7.07)	*z* = -.74 (*p* = .462)	*d* = .352
Degree of discomfort (0–10) [*M*(SD)]	2.20 (2.59)	2.53 (3.04)	*z* = -.05 (*p* = .960)	*d* = .116
Degree of control (0–10) [*M*(SD)]	6.98 (2.83)	7.53 (1.93)	*z* = -.38 (*p* = .702)	*d* = .227
Sexual intercourse with a steady partner	58.8% (n = 30)	62.3% (n = 43)	*χ*^2^ = .15 (*p* = .698)	*V* = .035
Time (minutes/week) [*M*(SD)]	130.71 (172.23)	71.09 (113.26)	*z* = -2.49 (*p* = .012)	*d*** = **.409
Orgasms (orgasms/week) [*M*(SD)]	1.93 (1.82)	2.27 (1.59)	*z* = -1.40 (*p* = .161)	*d* = .198
Degree of discomfort (0–10) [*M*(SD)]	.30 (1.06)	.83 (1.83)	*z* = -1.36 (*p* = .172)	*d* = .354
Degree of control (0–10) [*M*(SD)]	9.00 (1.99)	8.58 (1.66)	*z* = -1.44 (*p* = .149)	*d* = .149
Sexual intercourse with sporadic partners	11.8% (n = 6)	14.5% (n = 10)	*χ*^2^ = .19 (*p* = .664)	*V* = .040
Time (minutes/week) [*M*(SD)]	47.50 (50.57)	46.50 (41.50)	*z* = -.06 (*p* = .955)	*d* = .022
Orgasms (orgasms/week) [*M*(SD)]	1.33 (1.51)	1.29 (.95)	*z* = -.15 (*p* = .882)	*d* = .031
Degree of discomfort (0–10) [*M*(SD)]	2.17 (3.06)	4.00 (3.82)	*z* = -1.13 (*p* = .261)	*d* = .528
Degree of control (0–10) [*M*(SD)]	8.17 (1.83)	6.13 (3.91)	*z* = -.86 (*p* = .391)	*d* = .668

### Clinical Profile

The clinical profile of both groups was analyzed (Table 3). Starting with the variables related to OSA, we found statistically significant differences in the Internet Sex Screening Test (ISST). Individuals in the clinical group score higher on this scale than people in the subclinical group (*t* = -3.15; *p* = .002; *d* = .526). This difference in the total score seems obvious given that this was one of the criteria for assigning patients to the clinical vs. subclinical group. When comparing the scores of both groups in the different subscales, statistically significant differences in online social behavior are observed (*t* = − 3.550; *p* = .001). Individuals in the clinical group show a greater tendency to engage in interpersonal interactions with others during online sexual behavior than individuals in the subclinical group. The size of these differences is moderate (*d* = .526 and *d* = .652 respectively).

**Table 3:** Differences between subclinical and clinical (problematic) groups in clinical variables

	Subclinical group (n = 51) *M*(*SD)*	Clinical group (n = 69) *M*(*SD)*	*t* (*p*)	Effect size
Internet Sexual Screening Test	15.74 (3.17)	17.64 (3.32)	* − 3.15 (p* = .002)	*d* = .586
Online Sexual Compulsivity	5.04 (1.21)	5.17 (1.26)	− .58 (*p* = .558)	*d* = .105
Non-compulsive Solitary Online Behavior	4.86 (1.00)	5.17 (.86)	− 1.83 (*p* = .070)	*d* = .310
Social Online Behavior	3.02 (1.71)	4.10 (1.60)	− 3.55 (*p* = .001)	*d* = .652
Online Sexual Spending	.61 (.72)	.83 (.80)	− 1.53 (*p* = .128)	*d* = .289
Perceived Severity of Online Behavior	2.22 (.73)	2.36 (.71)	− 1.10 (*p* = .270)	*d* = .194
Sexual Sensation Seeking	25.51 (6.62)	30.39 (5.88)	− 4.26 (*p* < .001)	*d* = .781
Self-steem	28.59 (6.45)	27.07 (6.40)	1.27 (*p* = .204)	*d* = .237
State-Anxiety	25.72 (11.94)	31.29 (10.65)	− 2.67 (*p* = .009)	*d*= .506
Trait-Anxiesty	25.38 (11.55)	29.55 (9.24)	− 2.18 (*p* = .031)	*d* = .401
Depressive symptoms	14.86 (9.74)	18.67 (9.59)	− 2.12 (*p* = .036)	*d* = .394
Impulsivity	65.39 (7.07)	66.35 (7.31)	− .71 (*p* = .473)	*d* = .133
Dificulties in Emotional Regulation	69.37 (19.53)	70.83 (20.75)	− .38 (*p* = .698)	*d* = .072

Moreover, the differences between both groups in different clinical variables were also analyzed. Individuals in the clinical group presented higher levels of state anxiety (*M* = 31.29*; SD* = 5.88), trait anxiety (*M* = 29.55; *SD* = 9.24) and depression (*M* = 18.67; *SD* = 9.59) that individuals in the subclinical group (*M* = 25.72; *SD* = 11.94; *M* = 25.51; *SD* = 11.55: *M* = 14.86; *SD* = 9.74 respectively) (*p* between .009 and .036). In addition, individuals in the clinical group (*M* = 30.39; *SD* = 5.88) are also more sexual sensation seekers than individuals in the subclinical group (*M* = 25.51; *SD* = 6.62) (*p* < .001). These differences have a small to moderate effect size (*d* between .394 and .781). In contrast, no statistically significant differences were found in self-esteem, impulsivity and difficulties in emotional regulation.

### Predictive Variables of Problematic Engagement in Online Sexual Activities

To analyze which variables are related to the clinical profile, seven binary logistic regressions were carried out. In all of them, the backward stepwise method was used and belonging to the clinical group was incorporated as a dependent variable (DV) (0 = subclinical group, 1 = clinical group). First, time spent in each OSA and time in total OSA were included as independent variables (IV). The model was statistically significant (*χ*^*2*^ = 15.22; *df* = 6; *p* = .019) and presented a good fit overall (*χ*^*2*^ = 3.23; *df* = 8;* p* = .919). However, none of the variables included were significant (*p* between .069 and .098). In the subsequent regression, the time spent in offline sexual activities (total and of the different activities analyzed) were included as IV. The model was statistically significant (*χ*^*2*^ = 5.90; *df* = 1; *p* = .015) and presented a good fit overall *(χ*^*2*^ = 6.09; *df* = 5; *p* = .298). Nevertheless, as shown in Table 4, time spent in sexual intercourse with a steady partner was the only variable included in the final model, explaining 11.1% of the variance of our DV.

**Table 4:** Predictors of clinical profile related to time spent in offline sexual activities

							95% CI-Exp (B)	
	*β*	SE	Wald	*df*	*p*	*OR*	Lower	Upper
Constant	2.013	.377	28.450	1	< .001	7.484		
Time spent in sexual intercourse with a steady partner (minutes/week)	-.004	.002	5.851	1	.016	.996	.992	.999

At the four subsequent binary logistic regressions collected as IV (1) the orgasms from OSAs (*p* = .088); (2) the orgasms from offline sexual activities (*p* = .105); (3) degree of discomfort and control over the main OSAs (*p* = .281) and (4) degree of discomfort and control with offline sexual activities (*p* = .086), none of these models proved to be statistically significant. Detailed statistical results for the non-significant regression models are now available in the Supplementary Materials 5 to 9.

Finally, in the last regression, sexual sensation seeking, self-esteem, state anxiety and trait anxiety, depressive symptoms, impulsivity and difficulties in emotional regulation were included as independent variables. The model was statistically significant (*χ*^*2*^ = 26.63; *df* = 3; *p* < .001) and presented a good fit overall (*χ*^*2*^ = 7.08; *df* = 8; *p* = .528). As shown in Table 5, sexual sensation seeking, anxiety-trait and less difficulties in emotional regulation predicted 27.4% of the change in our DV, i.e., belonging to the clinical group.

**Table 5:** Clinical variables predictive of a clinical (problematic) profile

							95% CI-Exp (B)	
	*β*	SE	Wald	*df*	*p*	*OR*	Lower	Upper
Constant	− 3.612	1.211	8.896	1	.003	.027		
Sexual Sensation Seeking	.154	.039	15.644	1	< .001	1.166	1.081	1.258
Anxiety-Trait	.093	.033	8.095	1	.004	1.097	1.029	1.170
Dificulties in Emotional Regulation	− .041	.017	6.214	1	.013	.959	.929	.991

To assess the stability of these findings, robustness checks were performed using zero-order correlations and a hierarchical logistic regression (see Supplementary Tables 3 and 4). These analyses revealed a high degree of consistency with the primary results. Specifically, sexual sensation seeking (*r* = .36, *p* < .001) and trait anxiety (*r* = .19, *p* = .031) showed significant positive correlations with clinical group. Furthermore, the hierarchical model confirmed that sexual sensation seeking remains a robust predictor (*B* = .157, *p* < .001) even when accounting for sociodemographic factors. A minor difference noted was that, in the hierarchical model, total minutes online per week also emerged as a significant predictor (*B* = .001, *p* = .011).

## Discussion

The main aim of this study was to analyze the differences in sexual and clinical variables between individuals in subclinical and clinical groups of problematic engagement in OSA, as well as to identify factors related to the problematic engagement in OSA. Some differences were found between both groups in variables related to the way of performing each OSA and offline sexual activities, as well as in the presence of clinical symptomatology and other personality traits. Moreover, some predictor variables of a clinical profile were identified.

First, the differences between both groups in OSA were analyzed. Apparently, all participants in both groups watched pornographic images or films and a large number of respondents engaged in OSA in interaction with other users. However, in general, individuals in the clinical group spent significantly more time on these activities than those in the subclinical group. These data are similar to those found by Wetterneck et al. (2012). In addition, other authors such as Grov et al. (2008) also consider time as a relevant factor when identifying people with problematic engagement in OSA, establishing 10 h per week as a criterion that would indicate a problematic consumption. In our study, both subclinical and clinical users spent more than 10 h per week on average in these activities. These results would support this idea, although also highlight the need to take into account other factors when determining whether consumption is becoming problematic (subclinical) or already constitutes a clinical problem.

Regarding other variables related to these behaviors (orgasms experienced, degree of discomfort and degree of control), in general, individuals in the clinical group experienced more orgasms with these activities, as well as greater discomfort and a lower degree of control. However, statistically significant differences were only found in weekly orgasms experienced in the use of text-based sexual chats and webcam sexual contact. This difference in terms of orgasms experienced, which occur coincidentally in the sexual behaviors that cause the most discomfort in individuals in the clinical group, could be explained by the processes of tolerance and dissociation (Birchard, 2015; Love et al., 2015). One of the characteristics that people with problematic engagement in OSA may present is tolerance towards some sexual practices, which leads them to experience a progressive quantitative (i.e. increase in the time invested on pornography use) and qualitative escalation of their online sexual activity (progression to more diverse or extreme contents) (Ince et al., 2024). This, together with the dissociation that many people with problematic engagement in OSA experience during these sexual practices, would explain why they experience great pleasure from these riskier activities. But, once they become aware of what they have done, discomfort increases. Considering this possible explanation and the discrepancies regarding the presence of symptoms like tolerance in individuals experiencing difficulties with cybersex use, future research should employ more precise assessment techniques (such as longitudinal studies or the use of ecological momentary assessment. These methods would allow for more accurate information to be gathered about sexual behavior and the changes experienced during those moments, thus providing a clearer understanding of phenomena associated with tolerance, such as bingeing, edging, and tab-jumping (Ince et al., 2024; Lewczuk et al., 2022; Wordecha et al., 2018).

Regarding offline sexual activities, statistically significant differences were found only in the time spent in masturbation without online sexual material and in the sexual intercourse with a steady partner. People in the subclinical group spent more time on these activities than individuals in the clinical group. These differences between these two groups may be due, first, to the habituation to masturbate with online sexual material experienced by many problematic users, which markedly decreases the ability to achieve arousal outside of the Internet and without novel online sexual material (Bőthe et al., 2021; Love et al., 2015). However, neither these nor other studies specifically explore tolerance to pornography consumption in relation to a decreased ability to achieve arousal through masturbation without using the internet. Nevertheless, some data, such as those presented in this study, support the negative implications of this problem on sexual functioning. In fact, in the treatments used for online sexual compulsivity (aka, addiction or hypersexuality), one of the goals pursued is to promote healthy sexuality and healthy masturbation (Edwards et al., 2011; Schneider & Weiss, 2001; Ševčíková et al., 2018). Secondly, some authors such as Young et al. (2000) or Schneider (2000) suggest that people with problematic engagement in OSA experience sexual desire with OSAs, but show a decrease in their offline sexual desire, losing interest in having sex even with their own partner (Cavaglion, 2009; Schneider, 2000; Wright et al., 2019). This would explain why individuals in the clinical group dedicate fewer hours to these sexual behaviors. This is the only variable related to sexual behavior that has been found to be predictive of clinical profile. Specifically, spending more time on these sexual behaviors was negatively associated with problematic engagement in OSA, further reinforcing the need to promote healthy sexuality in problematic online sex use intervention programs. However, it must be taken into account that the manifestations of problematic engagement in OSA can be very diverse. For this reason, it is likely that we will not find great differences in these behaviors given the great diversity in the sexual behavior of both people who present a subclinical profile and those who present a clinical profile.

Moreover, we also explored the differences between both groups in the presence of clinical symptomatology and some personality-related factors. Participants in the clinical group in this study are more sexual sensation seekers than individuals in the subclinical group. In addition, this variable proved to be a predictor of the clinical profile. These data are similar to those obtained by Chen et al. (2018, 2019) or Walton et al. (2017) who also link the search for sexual sensations with cybersex consumption and highlight it as one of the key components in the problematic engagement in OSA. Regarding anxiety and depression, as evidenced by other studies, people with problematic engagement in OSA have a high comorbidity with mood and anxious disorders (Altin et al., 2024; Ballester-Arnal et al., 2020; Hermand et al., 2020; Vieira & Griffiths, 2024). Our data support this idea since individuals in the clinical group show higher levels of anxiety (state and trait) and depression. This could be explained by the hypothesis proposed by many authors suggesting that the use of sex is employed by these individuals as a strategy to regulate emotions. This strategy is reinforced by the temporary symptomatic relief that it produces. However, the symptomatology returns in a more exacerbated form due to the consequences caused by the excessive sexual behavior, so that emotional dysregulation would be a central component of sexual compulsivity/hypersexuality/sexual addiction because of its strong role in the maintenance of the symptoms (Hegbe et al., 2021; Lew-Starowicz et al., 2020; Rahm-Knigge et al., 2023; Schultz et al., 2014). In this sense, our participants also obtained relatively high scores for difficulties in emotional regulation, which is further evidence for this hypothesis. Not all clinical variables related to negative emotional states turned out to be predictors of the clinical profile. Specifically, only state anxiety predicted the clinical profile. This may be due to the relevant role that more stable traits such as trait-anxiety may have in the creation and maintenance of the problem. Regarding difficulties in emotional regulation, contrary to what we expected, in our study it is negatively related to the clinical profile. This could be due to the few differences we found between both groups in this variable. Another possible explanation is related to the nature of the DERS as a self-report measure. Since it requires introspection and emotional awareness, individuals in the clinical group—especially in early stages of treatment—may have limited insight or willingness to recognize their emotional difficulties, leading to an underestimation of their regulation problems. Therefore, further studies and other measures (e.g. studying exactly the different types of emotional regulation) would be needed to test these hypotheses.

Contrary to what is evidenced by some studies (Antons & Brand, 2018; Borgogna et al., 2020; Hegbe et al., 2021; Kor et al., 2014; Wéry et al., 2020) in our study neither self-esteem nor impulsivity turned out to be predictors of problematic engagement in OSA. Furthermore, no statistically significant differences were found in these variables between the two groups. One of the possible explanations may be due to the fact that some of these studies use non-clinical samples or samples that have not been thoroughly evaluated. Another reason may be related to the profile of our participants. In both groups, the mean scores obtained by the participants evidenced self-esteem (scores > 25) and moderate impulsivity (scores between 52 and 71). Moreover, when identifying subjects with behavioral addictions (e.g., pathological gambling) this questionnaire does not present as much sensitivity (Reid et al., 2014; Rodriguez-Jimenez et al., 2006) which could have biased these results. To address these issues, future studies should develop specific measures of sexual impulsivity and compulsivity that include specific aspects of these dimensions associated with sexual compulsivity (Benucci et al., 2024; Gaudet et al., 2025).

In short, as we have been able to observe throughout this study, it seems that individuals in the clinical group show some important differences in comparison with individuals in the subclinical group, which could help us to improve knowledge about this pathology, as well as the identification of diagnostic criteria. In addition, some predictive variables of clinical profile have also been identified, which could also help to improve the prevention of these problems.

### Limitations and Future Directions

This study had some limitations. One of them had to do with the particularities of this disorder and the difficulties we found when making a diagnosis. Since there is no consensus regarding its diagnostic criteria, it is difficult to make an accurate diagnosis of this pathology and even more to differentiate people who have a subclinical profile from those who have a clinical profile. Furthermore, the lack of consensus regarding the best approach for the diagnosis of out-of-control sexual behavior led us to include multiple diagnostic proposals (in particular, the compulsive sexual behavior disorder criteria, the sexual addiction criteria, and the hypersexual disorder criteria) for the identification of clinical patients. In our opinion, this approach lets to capture the wide diversity of sexual symptoms displayed by individuals with an excessive and problematic engagement in online sexual activities. For example, the criteria derived from the addiction diagnostic framework emphasize tolerance and withdrawal as symptoms of problematic engagement in OSAs; while some consider these symptoms relevant when it comes to diagnose this condition (Sassover & Weinstein, 2021), some researchers such as Billieux et al. (2015) and more recently Grubbs and Boness (2025) have denounced that behavioral addiction research is markedly flawed because of its uncritical adoption of substance use disorder frameworks and methods, which results in an erroneous understanding of these phenomena. Kafka’s (2010) proposal of hypersexual disorder as a sexual disorder proposed a set of criteria that were then supported by a field trial (Reid et al., 2012), emphasizing sexual behavior as an emotional regulation response (Castro-Calvo et al., 2020) but the DSM-5 Board of Trustees of the American Psychiatric Association finally decided to reject its inclusion (Kafka, 2014). More recently, the recognition of CSBD in the ICD-11 has strengthened its clinical legitimacy (Kraus et al., 2018), although debate persists as to whether it is best conceptualized as an impulse-control disorder, an addiction, or as part of another spectrum (Bothe et al., 2022). Considering these limitations, our approach based on the use of multiple diagnostic criteria may improve the clinical diagnosis of individuals struggling with sexual problems as it provides a solid foundation for the identification of the condition across diagnostic frameworks. To our knowledge, this is the only study employing such a comprehensive assessment and considering all existing diagnostic criteria in parallel, which provides more confidence in the validity of the diagnosis. Our study may also contribute to improving the diagnosis of this condition and, together with future research, help establish diagnostic criteria for problematic cybersex use.

In addition, we did not include widely used instruments for the assessment of out-of-control sexual behavior for classification of the sample, such as the SAST (Carnes, 1989) or the SCS (Kalichman & Rompa, 1995). These scales are conceived from different theoretical perspectives and do not provide cut-offs that distinguish between at-risk and clinical profiles. Instead, we employed the ISST because it is the only instrument that provides validated cut-off points to distinguish recreational, at-risk, and problematic profiles. Incorporating additional measures in future research could enrich the assessment and provide a more comprehensive picture of problematic engagement in OSA. Furthermore, future studies may include alternative methodological approaches to delineate subclinical and clinical groups. In particular, clustering techniques such as latent class analysis could be particularly useful for advancing classification systems through a data-driven approach.

Another limitation of this study relates to the statistical approach used. While stepwise regression analyses can be useful in exploratory contexts—particularly in emerging research areas where theoretical guidance is still limited—they also have methodological limitations. To mitigate these issues, we conducted supplementary analyses—zero-order correlations and a hierarchical logistic regression—which produced comparable results and increased confidence in the robustness of our findings. Nevertheless, future studies should replicate these analyses using larger samples and using other more robust analytical methods.

The sociodemographic characteristics of clinical samples with out-of-control online sexual activity also limit the generalizability of the findings. In addition to the gender imbalance, our sample consisted mainly of young adults and individuals with a relatively high educational level. Whereas in clinical settings this is the more typical patients’ profile (thus supporting the clinical representativity of our sample), this may also restrict the generalizability of our data to other populations (e.g., women or gender-diverse). Future studies should include more heterogeneous samples in terms of age, educational background, and gender. Conducting studies specifically focused on women, as well as on diverse populations, will be crucial to better define and understand subclinical and clinical profiles of engagement in OSA.

Beyond methodological and sampling considerations, our results also have potential clinical implications. Identifying differential characteristics between clinical and subclinical profiles may help clinicians to better recognize individuals at risk, improving our diagnostic approaches and designing more targeted interventions. In addition, one of the potential contributions of this study is that, within the same sample, we explored the presence or absence of all the symptoms and diagnostic criteria proposed in the literature. This comprehensive approach may be useful to identify which specific criteria or symptoms are most relevant when distinguishing between clinical and subclinical profiles. Taken together, these findings may ultimately contribute to refining current diagnostic criteria and informing clinical decision-making in the evaluation and treatment of problematic OSA.

## Supplementary Information

Below is the link to the electronic supplementary material.

Supplementary file1 (DOCX 28 KB)

Supplementary file2 (DOCX 29 KB)
