# Supplementary material for: Sexual and Clinical Profile in Spanish-Speaking Individuals with Problematic Engagement in Online Sexual Activities: Comparison Between Subclinical and Clinical Groups
Source: Arch Sex Behav. 2026 Jun 1;55(4):1661–78. doi: 10.1007/s10508-026-03434-0 (PMC13275794; doi:10.1007/s10508-026-03434-0)
Supplement: Supplementary file 1 — Supplementary file1 (DOCX 28 KB) [file 10508_2026_3434_MOESM1_ESM.docx]

| **Supplementary table 1**  *Frequencies and mean scores of online sexual activities and related variables performed by less than 10% of the sample in subclinical and clinical groups.* | | |
| --- | --- | --- |
|  | Subclinical group (n=51) | Clinical group (n=69) |
| Online Sexual Activities |  |  |
| Serch for a romantic partner | 0% (n=0) | 7.2% (n=5) |
| Time (minutes/week) [*M*(SD)] | __^a^ | 4.78 (29.93) |
| Orgasms (orgasms/week) [*M*(SD)] | __^a^ | 1.00 (2.00) |
| Degree of discomfort (0-10) [*M*(SD)] | __^a^ | 3.50 (2.65) |
| Degree of control (0-10) [*M*(SD)] | __^a^ | 6.75 (1.26) |
| Purchase of sexual material online | 5.9% (n=3) | 4.3% (n=3) |
| Time (minutes/week) [*M*(SD)] | .39 (2.80) | 5.22 (36.73) |
| Orgasms (orgasms/week) [*M*(SD)] | 0 (0.00) | 1.00 (1.41) |
| Degree of discomfort (0-10) [*M*(SD)] | 5.00 (4.07) | 8.00 (1.41) |
| Degree of control (0-10) [*M*(SD)] | 5.00 (4.07) | 6.00 (3.24) |
| Contact with sex workers | 9.8% (n=5) | 8.7% (n=6) |
| Time (minutes/week) [*M*(SD)] | 1.27 (5.90) | 13.48 (52.10) |
| Orgasms (orgasms/week) [*M*(SD)] | 0.50 (0.57) | 1.43 (1.27) |
| Degree of discomfort (0-10) [*M*(SD)] | 8.25 (1.71) | 6.14 (3.29) |
| Degree of control (0-10) [*M*(SD)] | 7.50 (3.11) | 3.43 (2.29) |
| Note: __^a^= empty cells correspond to data not provided because the number of participants who engaged in that behavior was 0. | | |

| **Supplementary table 2**  *Frequencies and mean scores of offline sexual activities and related variables performed by less than 10% of the sample in subclinical and clinical groups.* | | |
| --- | --- | --- |
|  | Subclinical group (n=51) | Clinical group (n=69) |
| Offline Sexual Activities |  |  |
| Sexual intercourse with people other than one’s partner (infidelity) | 3.9% (n=2) | 7.2% (n=5) |
| Time (minutes/week) [*M*(SD)] | 60.00 (84.85) | 45.00 (47.43) |
| Orgasms (orgasms/week) [*M*(SD)] | 1.00 (1.41) | 1.00 (1.00) |
| Degree of discomfort (0-10) [*M*(SD)] | 7.75 (3.18) | 7.00 (4.12) |
| Degree of control (0-10) [*M*(SD)] | 6.50 (4.95) | 2.20 (2.28) |
| Sexual intercourse with sex workers | 2% (n=1) | 8.7% (n=6) |
| Time (minutes/week) [*M*(SD)] | 0 (.00) | 52.50 (70.83) |
| Orgasms (orgasms/week) [*M*(SD)] | 0 (.00) | 1.80 (1.92) |
| Degree of discomfort (0-10) [*M*(SD)] | 10.00 (.00) | 6.50 (3.88) |
| Degree of control (0-10) [*M*(SD)] | 7.00 (.00) | 1.67 (1.97) |

| **Supplementary table 3**  *Zero-order correlations of the variables included in the different regressions* | | |
| --- | --- | --- |
| Group (0=subclinical; 1=clinical) | | |
| **Minutes online/week** | ***r* (*p*)** | **.231 (.011)** |
| Viewing pornographic images or films (minutes/week) | *r* (*p*) | .165 (.072) |
| Viewing pornographic images or films (orgasms) | *r* (*p*) | .089 (.346) |
| Viewing pornographic images or films (discomfort) | *r* (*p*) | .059 (.529) |
| Viewing pornographic images or films (control) | *r* (*p*) | -.014 (.879) |
| **Flirting and sexual advances towards other users (minutes/week)** | *r* (*p*) | **.236 (.039)** |
| Flirting and sexual advances towards other users (orgasms) | *r* (*p*) | .181 (.122) |
| Flirting and sexual advances towards other users (discomfort) | *r* (*p*) | .098 (.394) |
| Flirting and sexual advances towards other users (control) | *r* (*p*) | -.172 (.129) |
| Chatting for sexual purposes with other users via text (minutes/week) | *r* (*p*) | .178 (.120) |
| **Chatting for sexual purposes with other users via text (orgasms)** | *r* (*p*) | **.281 (.013)** |
| Chatting for sexual purposes with other users via text (discomfort) | *r* (*p*) | .089 (.432) |
| Chatting for sexual purposes with other users via text (control) | *r* (*p*) | -.141 (.210) |
| Sexual contact via webcam with another user (minutes/week) | *r* (*p*) | .222 (.085) |
| **Sexual contact via webcam with another user (orgasms)** | *r* (*p*) | **.312 (.015)** |
| Sexual contact via webcam with another user (discomfort) | *r* (*p*) | .027 (.837) |
| Sexual contact via webcam with another user (control) | *r* (*p*) | -.038 (.771) |
| Search for a sexual partner (minutes/week) | *r* (*p*) | .171 (.342) |
| Search for a sexual partner (orgasms) | *r* (*p*) | .140 (.459) |
| Search for a sexual partner (discomfort) | *r* (*p*) | -.208 (.260) |
| Search for a sexual partner (control) | *r* (*p*) | -.210 (.249) |
| Minutes offline/week | *r* (*p*) | .051 (.580) |
| Masturbation without online sexual material (minutes/week) | *r* (*p*) | -.165 (.116) |
| Masturbation without online sexual material (orgasms) | *r* (*p*) | .162 (.247) |
| Masturbation without online sexual material (discomfort) | *r* (*p*) | .054 (.688) |
| Masturbation without online sexual material (control) | *r* (*p*) | .117 (.383) |
| **Sexual intercourse with a steady partner (minutes/week)** | *r* (*p*) | **-.202 (.048)** |
| Sexual intercourse with a steady partner (orgasms) | *r* (*p*) | .101 (.424) |
| Sexual intercourse with a steady partner (discomfort) | *r* (*p*) | .168 (.160) |
| Sexual intercourse with a steady partner (control) | *r* (*p*) | -.115 (.341) |
| Sexual intercourse with sporadic partners (minutes/week) | *r* (*p*) | -.012 (.966) |
| Sexual intercourse with sporadic partners (orgasms) | *r* (*p*) | -.021 (.946) |
| Sexual intercourse with sporadic partners (discomfort) | *r* (*p*) | .268 (.354) |
| Sexual intercourse with sporadic partners (control) | *r* (*p*) | -.322 (.262) |
| **Internet Sexual Screening Test - ISST** | *r* (*p*) | **.279 (.002)** |
| **Sexual Sensation Seeking (SSSS)** | *r* (*p*) | **.365 (<.001)** |
| Self-steem (RSEI) | *r* (*p*) | -.117 (.204) |
| **State-Anxiety (STAI-S)** | *r* (*p*) | **.240 (.009)** |
| **Trait-Anxiesty (STAI-T)** | *r* (*p*) | **.198 (.031)** |
| **Depressive symptoms (BDI)** | *r* (*p*) | **.192 (.036)** |
| Impulsivity (BISS-II) | *r* (*p*) | .066 (.473) |
| Dificulties in Emotional Regulation (DERS) | *r* (*p*) | .036 (.698) |

| **Supplementary table 4**  *Hierarchical logistic regression predicting clinical profile of problematic engagement in OSA.* | | | | | | | | |
| --- | --- | --- | --- | --- | --- | --- | --- | --- |
|  | B | S.E. | Wald | df | *p*. | Exp(B) | 95% C.I.for EXP(B) | |
|  |  |  |  |  |  |  | Lower | Upper |
| Sex | -.210 | 1.092 | .037 | 1 | .848 | .811 | .095 | 6.897 |
| Age | .000 | .024 | .000 | 1 | .995 | 1.000 | .955 | 1.047 |
| **Sexual Sensation Seeking (SSSS)** | **.157** | **.042** | **13.651** | **1** | **.000** | **1.170** | **1.076** | **1.271** |
| State-Anxiety (STAI-S) | .048 | .030 | 2.580 | 1 | .108 | 1.049 | .990 | 1.111 |
| Trait-Anxiesty (STAI-T) | .052 | .042 | 1.501 | 1 | .220 | 1.053 | .969 | 1.145 |
| Depressive symptoms (BDI) | .013 | .038 | .111 | 1 | .739 | 1.013 | .940 | 1.091 |
| Impulsivity (BISS-II) | .008 | .032 | .063 | 1 | .802 | 1.008 | .947 | 1.074 |
| **Dificulties in Emotional Regulation (DERS)** | **-.056** | **.021** | **7.393** | **1** | **.007** | **.946** | **.908** | **.984** |
| **Minutes online/week** | **.001** | **.000** | **6.478** | **1** | **.011** | **1.001** | **1.000** | **1.002** |
| Self-steem (RSEI) | -.012 | .048 | .066 | 1 | .797 | .988 | .899 | 1.086 |
| Constant | -4.031 | 3.210 | 1.577 | 1 | .209 | .018 |  |  |
| Note. Model fit: Omnibus Test *χ²*(8) = 39.38, *p* < .001; Hosmer–Lemeshow *χ²* = 13.70, *p* = .090; Nagelkerke R² = .385. | | | | | | | | |
