# Supplementary material for: Sexual and Clinical Profile in Spanish-Speaking Individuals with Problematic Engagement in Online Sexual Activities: Comparison Between Subclinical and Clinical Groups
Source: Arch Sex Behav. 2026 Jun 1;55(4):1661–78. doi: 10.1007/s10508-026-03434-0 (PMC13275794; doi:10.1007/s10508-026-03434-0)
Supplement: Supplementary file 2 — Supplementary file2 (DOCX 29 KB) [file 10508_2026_3434_MOESM2_ESM.docx]

| **Supplementary table 5**  *Results for the non-significant predictors in the logistic regression model of time spent in Online Sexual Activities (OSA) (Model 1).* | | | | | | | |
| --- | --- | --- | --- | --- | --- | --- | --- |
|  |  |  |  |  |  | 95% CI-Exp (B) | |
|  | *β* | SE | Wald | *df* | *p* | Lower | Upper. |
| Constant | -3.826 | 2.453 | 2.433 | 1 | .119 |  |  |
| Time spent on OSAs in general | .162 | .091 | 3.153 | 1 | .076 | .983 | 1.407 |
| Time spent on viewing pornographic images or films | -.164 | .092 | 3.160 | 1 | .075 | .709 | 1.017 |
| Time spent on flirting and sexual advances towards other users | -.165 | .092 | 3.232 | 1 | .072 | .708 | 1.015 |
| Time spent on chatting for sexual purposes with other users via text | -.149 | .090 | 2.737 | 1 | .098 | .721 | 1.028 |
| Time spent on sexual contact via webcam with another user, search for a sexual partner | -.155 | .089 | 3.009 | 1 | .083 | .719 | 1.020 |
| Time spent on search for a sexual partner | -.166 | .091 | 3.302 | 1 | .069 | .708 | 1.013 |

| **Supplementary table 6**  *Results for the non-significant predictors in the logistic regression model of orgasms experienced in OSA (Model 3).* | | | | | | | |
| --- | --- | --- | --- | --- | --- | --- | --- |
|  |  |  |  |  |  | 95% CI-Exp (B) | |
|  | *β* | SE | Wald | *df* | *p* | Lower | Upper. |
| Constant | .042 | 1.205 | .001 | 1 | .972 |  |  |
| Orgasms experienced in viewing pornographic images or films | -.131 | .157 | .702 | 1 | .402 | .645 | 1.192 |
| Orgasms experienced in flirting and sexual advances towards other users | -.497 | .521 | .910 | 1 | .340 | .219 | 1.689 |
| Orgasms experienced in chatting for sexual purposes with other users via text | 1.019 | .827 | 1.520 | 1 | .218 | .548 | 13.999 |
| Orgasms experienced in sexual contact via webcam with another user, search for a sexual partner | .262 | .198 | 1.746 | 1 | .186 | .881 | 1.915 |
| Orgasms experienced in search for a sexual partner | -.547 | .606 | .814 | 1 | .367 | .177 | 1.897 |

| **Supplementary table 7**  *Results for the non-significant predictors in the logistic regression model of orgasms experienced in offline sexual activities (Model 4).* | | | | | | | |
| --- | --- | --- | --- | --- | --- | --- | --- |
|  |  |  |  |  |  | 95% CI-Exp (B) | |
|  | *β* | SE | Wald | *df* | *p* | Lower | Upper. |
| Constant | -.382 | .713 | .287 | 1 | .592 |  |  |
| Orgasms experienced in masturbation without online sexual material | .079 | .158 | .253 | 1 | .615 | .795 | 1.475 |
| Orgasms experienced in sexual intercourse with steady partners | .072 | .202 | .128 | 1 | .721 | .723 | 1.597 |
| Orgasms experienced in sexual intercourse with sporadic partners* |  |  |  |  |  |  |  |
| Note: *variables were excluded from the final regression models due to an insufficient number of valid cases. | | | | | | | |

| **Supplementary table 8**  *Results for the non-significant predictors in the logistic regression model of discomfort and control over the different OSAs (Model 5).* | | | | | | | |
| --- | --- | --- | --- | --- | --- | --- | --- |
|  |  |  |  |  |  | 95% CI-Exp (B) | |
|  | *β* | SE | Wald | *df* | *p* | Lower | Upper. |
| Constant | 1.220 | 1.753 | .483 | 1 | .487 |  |  |
| Degree of control on viewing pornographic images or films | .200 | .157 | 1.625 | 1 | .202 | .898 | 1.659 |
| Degree of discomfort on viewing pornographic images or films | -.080 | .191 | .176 | 1 | .675 | .635 | 1.342 |
| Degree of control on flirting and sexual advances towards other users | -.182 | .314 | .339 | 1 | .561 | .451 | 1.541 |
| Degree of discomfort on flirting and sexual advances towards other users | -.161 | .261 | .377 | 1 | .539 | .510 | 1.422 |
| Degree of control on chatting for sexual purposes with other users via text | -.069 | .399 | .030 | 1 | .862 | .427 | 2.038 |
| Degree of discomfort on chatting for sexual purposes with other users via text | -.019 | .267 | .005 | 1 | .943 | .581 | 1.656 |
| Degree of control on sexual contact via webcam with another user, search for a sexual partner | .104 | .230 | .204 | 1 | .651 | .707 | 1.742 |
| Degree of discomfort on sexual contact via webcam with another user, search for a sexual partner | .021 | .192 | .012 | 1 | .915 | .700 | 1.489 |
| Degree of control on search for a sexual partner* |  |  |  |  |  |  |  |
| Degree of discomfort on search for a sexual partner* |  |  |  |  |  |  |  |
| Note: *variables were excluded from the final regression models due to an insufficient number of valid cases. | | | | | | | |

| **Supplementary table 9**  *Results for the non-significant predictors in the logistic regression model of time spent in*  *Offline Sexual Activities (Model 6).* | | | | | | | |
| --- | --- | --- | --- | --- | --- | --- | --- |
|  |  |  |  |  |  | 95% CI-Exp (B) | |
|  | *β* | SE | Wald | *df* | *p* | Lower | Upper. |
| Constant | .478 | 1.642 | .085 | 1 | .771 |  |  |
| Degree of control on offline sexual activities in general | -.051 | .156 | .107 | 1 | .743 | .700 | 1.290 |
| Degree of discomfort on offline sexual activities in general | .183 | .213 | .739 | 1 | .390 | .791 | 1.825 |
| Degree of control on masturbation without online sexual material | .520 | .379 | 1.888 | 1 | .169 | .801 | 3.532 |
| Degree of discomfort on masturbation without online sexual material | -.231 | .228 | 1.029 | 1 | .310 | .508 | 1.240 |
| Degree of control on sexual intercourse with sporadic partners* |  |  |  |  |  |  |  |
| Degree of discomfort on sexual intercourse with sporadic partners* |  |  |  |  |  |  |  |
| Note: *variables were excluded from the final regression models due to an insufficient number of valid cases. | | | | | | | |
